# Single gametophyte sequencing reveals that crossover events differ between sexes in maize

**DOI:** 10.1038/s41467-019-08786-x

**Published:** 2019-02-15

**Authors:** Cheng Luo, Xiang Li, Qinghua Zhang, Jianbing Yan

**Affiliations:** National Key Laboratory of Crop Genetic Improvement, Huazhong Agricultural University, Wuhan, 430070 China

## Abstract

Meiotic crossover (CO) plays a key role in producing gametophytes and generating genetic variation. The patterns of CO production differ inter- and intra-species, as well as between sexes. However, sex-specific patterns of CO production have not been accurately profiled independently of genetic backgrounds in maize. Here, we develop a method to isolate single female gametophyte for genomes sequencing in maize. We show that more COs are observed in male (19.3 per microspore) than in female (12.4 per embryo sac). Based on Beam-Film model, the more designated class I and II COs are identified in male than in female. In addition, CO maturation inefficiency (CMI) is detected in some genetic backgrounds, suggesting that maize may be an ideal model for dissecting CMI. This research provides insights toward understanding the molecular mechanism of CO production between sexes and may help to improve maize breeding efficiency through paternal selection.

## Abstract (web summary)

Meiotic crossover (CO) landscape differs inter- and intra-species, as well as between sexes. Here, the authors show that male meiosis produces more COs than female in maize and detect CO maturation inefficiency in some genetic backgrounds, which may help to improve breeding efficiency.

## Introduction

Meiosis transforms sexual organisms from sporophyte (2*n*) to gametophyte (*n*) generation. Crossover (CO) is a key event during meiosis, since it not only ensures the success of homologous chromosome separation by chiasmata^1,2^ but also introduces large-scale genetic variation by inter-homolog exchange. CO is produced in a series of highly regulated steps. Briefly, meiotic recombination initiates from double-strand breaks (DSBs) followed by inter-homolog invasion, generating inter-homologous intermediates, which will be resolved to CO or non-crossover (NCO) recombination through different pathways, including double Holliday junction and synthesis-dependent strand annealing^3–5^. CO frequency, distribution, and interference could differ inter-^6^ and intra-species^7^. Sex differences in CO frequency was discovered first in the 1920s^8,9^, and has been observed in many species to date^10^. In fruit fly^11^ and silkworm^12^, in particular, COs were produced only in male and female meiosis, respectively. Many factors contribute to sex difference in CO production. First, given that synaptonemal complex (SC) length was positively correlated with CO amount^13,14^, the sex with longer SC harbors more COs, such as female of human^15^ and mouse^16^. Second, it was reported that 13 variants were identified as candidates to affect recombination rate in the Icelandic human population. Three of them only affect the male recombination rate, and seven of them only affect the female recombination rate^17^. These suggested that the sex-specific variants could result in divergence of CO pattern, probably through governing meiotic DSB initiation and repair pathway. Third, selfish elements increase their transmission probability during sex-specific gametogenesis, often disrupting the Mendelian segregation ratio (2:2) in only one sex, like t-haplotype for male in mouse^18^ and abnormal chromosome 10 (Ab10) for female in maize^19,20^.

Dioecious species, such as human and mouse, may not be perfect models to estimate sex difference of CO production, since the difference between individuals is derived not only from sexes but also from genetic backgrounds. Hermaphroditic species, like *Arabidopsis* and maize, can produce the gametophytes sharing the same background; in this case, the difference in CO pattern could only result from sex-specific elements. In *Arabidopsis* and maize, genotyping individuals from reciprocal crosses revealed sex differences in CO patterns^21–23^. Comparison of CO patterns between single sperm and egg^24,25^ in human provides a strategy to evaluate sex differences of recombination pattern during one meiosis. In maize, single microspores were isolated and sequenced to profile the male recombination pattern^26^. Although the isolation of maize embryo sacs and eggs was reported^27^, sequencing remains a challenge, owing to the large number of nucellar cells around embryo sac. Here we develop a method to isolate antipodal cells from single embryo sacs for DNA amplification and high-throughput sequencing. The female CO pattern is profiled in high resolution. Through comparing the CO patterns of embryo sac and microspore populations sharing the same background, sex differences are found, with fewer COs and hot regions in female than male. The 84% difference in CO number is attributed to the difference in the number of CO hot regions between sexes. Based on Beam-Film (BF) model, it is determined that longer interference distance and fewer non-interfering COs (class II COs) are in female, independent of genetic backgrounds. CO maturation inefficiency (CMI) exists in some genetic backgrounds. These results provide insights into the mechanism of sex-specific CO production and toward improving breeding efficiency in maize. Single gametophyte sequencing could be a general strategy to analyze sex-specific recombination among any species.

## Results

### Isolation of antipodal cells from a single embryo sac

**Fig. 1:**
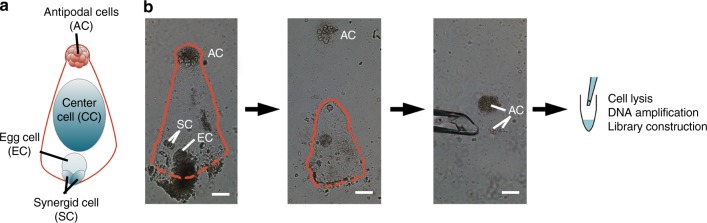
The microscopic isolation of antipodal cells from maize embryo sac. **a** A diagram of a mature embryo sac. **b** The brief steps of microscopic isolation. The red line profiles embryo sac putatively. Bar = 100 μm

In angiosperm, female gametophyte generation begins at meiosis, which triggers megaspores from a megasporocyte. Micropylar megaspores are degraded, and the chalazal-most megaspore divides producing an embryo sac^28^. To isolate the female gametophyte, F_1_ individuals were planted. Their background (Zheng58 × SK) was the same as those of the microspore-derived F_1_ individuals sequenced previously^26^. A mass of cells, including embryo sac and nucellar cells, was extracted from the ovule and put into a drop of multiple enzymatic mixture for 15 min. The embryo sac could then be distinguished, separated from nucellar cells using a glass micropipette, and transferred to a clear drop of isotonic buffer. In *Arabidopsis*, one embryo sac contains three antipodal cells; however, dozens of antipodal cells were reported in maize^29^ (Fig. 1a). These antipodal cells share the same haplotype. To increase the copy number of the original DNA for amplification and sequencing coverage, a few (5–15) antipodal cells were isolated from the same embryo sac for cell lysis and multiple displacement amplification (MDA) of DNA (see Methods; Fig. 1b). The integrity of MDA products was validated using 10 molecular markers as described previously^26^. The amplified products with at least 8 positive markers could be qualified (Supplementary Figure 1) for sequencing. In total, 106 samples of qualified embryo sac DNA from 14 F_1_ individuals were selected for whole-genome sequencing. A total of 3,009,050,118 reads were obtained. On average, 28,215,923 filtered reads per sample could be mapped to and covered ~40% of the maize B73 v4 reference genome^30^ at ~1.2× depth (Supplementary Data 1). Based on the fine-assembled B73 v4 reference genome and validation results, the invalidated small bins and false-positive COs have been eliminated for further analysis (for details see Methods). To compare CO landscapes between both sexes, the same standard was also applied to the single microspore (*n* = 96) sequencing reads^26^. The male reads were re-mapped with ~29% of coverage, at 1.0× depth on average, lower than before, because the present analysis process is stricter and more reliable (see Methods). The higher genome coverage of female than male reads could be explained by a number of original antipodal cells (DNA copies, see above). In total, 3,799,510 raw single-nucleotide polymorphisms (SNPs) were called between parents. For female, 2,033,416 high-quality SNPs were obtained, with an average of 1,411,764 SNPs per sample; for male, 1,891,537 high-quality SNPs were obtained with average of 975,349 SNPs per sample (Supplementary Table 1, see Methods). The median distances of adjacent high-quality SNPs in female and male are 73 and 71 bp (Supplementary Figure 2), respectively. Approximately 90% of the adjacent high-quality SNPs are <1 kb apart (Supplementary Figure 2), which represents an extremely high marker resolution. This suggests that a pipeline based on the improved B73 V4 reference is more accurate.

### CO detection

**Fig. 2:**
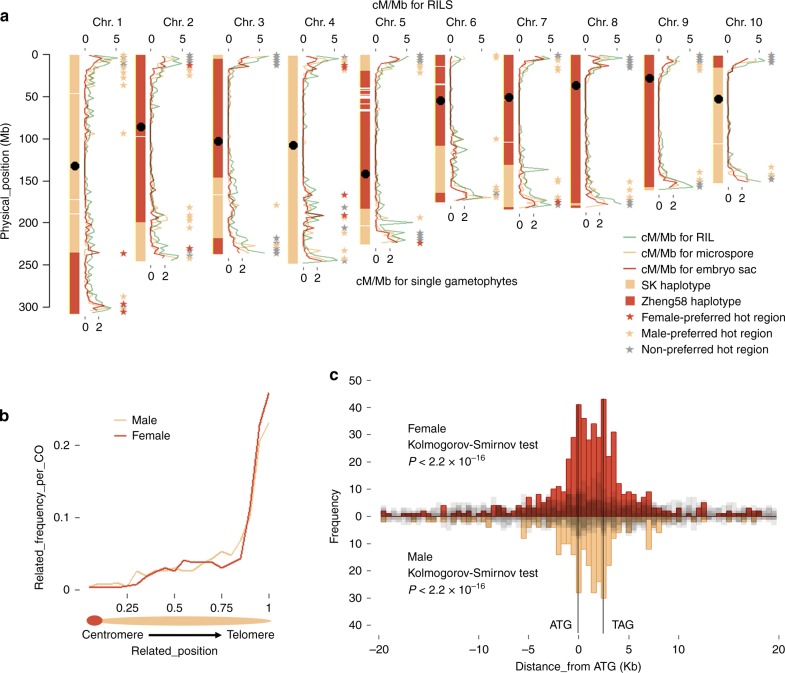
Sex-dimorphic CO landscape detected by single gametophyte sequencing. **a** For each chromosome, CO pattern includes three columns: the bin map of embryo sac 83 (left); CO distribution (cM/Mb) of microspore, embryo sac, and RIL populations (median); and hot region distribution (right). In the bin map (left), each horizontal line represents parental SNP. The column with consecutive lines suggest a high resolution of SNP. **b** The normalized CO frequency of male and female gametophytes was shown along chromosome arm. **c** The histograms show distance from each CO to the closest gene of the female (red) and male (yellow) gametophyte sets, and five simulated populations (gray)

For each sample, these SNPs were distributed evenly along chromosomes (Fig. 2a). In the microspore (M33, containing 485k SNPs) and embryo sac (E4, containing 520k SNPs) samples with the lowest coverage (14.9% and 15.8%, respectively), not any distinct gaps were detected (Supplementary Figure 3), suggesting that these datasets could be used to detect the COs perfectly. With high SNP density, large marker haplotype segments ending with the opposite parental haplotype could be defined as the preliminary bins. Bins sharing the same positions in several individuals could have been derived from residual heterozygous regions of parents and were removed. Sanger sequencing was performed to validate all the identified short bins (*n* = 28, <1.2 Mb), in which four bins were validated as false positive and 12 could not be validated as positive or false positive due to technique problem. Finally, the 16 short bins were removed for further analysis (Supplementary Table 2). The junctions of two adjacent bins could be taken as COs. Consequently, 1312 and 1856 COs were identified in 106 embryo sacs and 96 microspores, respectively (Supplementary Data 2). Female CO numbers range from 6 to 19, in average 12.4 per embryo sac; and male CO numbers range from 9 to 29, in average 19.3 per microspore (Supplementary Data 3). CO frequency in female (~0.29 cM/Mb, total 614.8 cM) is 0.64-fold of CO frequency in male (~0.46 cM/Mb, total 962.5 cM). In human^15^ and mouse^16^, more COs occur in female than male, which likely results from longer SC length for female. In *Arabidopsis*^21^, female meiosis produces less COs along a shorter SC than male. However, owing to that synaptene of maize female meiosis was hardly captured, sex difference of SC length has not been profiled in maize until now. Additionally, it was also observed that female CO numbers are positively correlated with chromosome length (physical length and male SC length of the inbred line KYS^14^) (Supplementary Figure 4), in accord with previous results in male^26^. CO frequency from the 14 embryo sac donors was relatively stable (Supplementary Figure 5), while the two microspore donors produced different CO amounts^26^.

### Sex-specific CO distribution

The recombination frequency map (cM/Mb) was constructed using 3 Mb windows for each gametophyte population and was compared to the map for recombinant inbred line (RIL) population sharing the same genetic background^31^ (Fig. 2a). To accurately estimate the chromosome-level difference between sexes, position of each CO was divided by chromosome arm length. Both male and female COs were concentrated in peri-telomere with the coincided distribution patterns (Fig. 2b). This was inconsistent within human that male meiosis produces more COs in peri-telomere than female, although COs in both sex were concentrated in peri-telomere^32^. The gametophyte and RIL populations within the same background share a similar CO distribution pattern (Fig. 2a). It was also reported that different CO distribution patterns were uncovered in populations with different genetic backgrounds^7^. These suggest that genetic background may affect CO distribution, probably through the genome modifications or (and) hotspot distribution. To further compare the sex-specific CO patterns, CO hot regions were defined as the 3 Mb windows with more than 1 cM/Mb and a total of 123 hot regions were discovered (stars in Fig. 2a, Supplementary Data 4). In female and male, 71 (10.1%) and 112 (15.9%) hot regions contained 755 (57.55%) and 1190 (64.12%) COs, respectively, which suggests that the dramatic difference in CO frequency between sexes was positively correlated by the differing number of hot regions. To further profile difference of hot regions between gametophyte populations, sex-preferred hot regions were defined as greater than twofold CO frequency of the other sex. We observed 11 female-preferred (red stars in Fig. 2a) and 53 male-preferred (yellow stars in Fig. 2a) hot regions. One of the extreme male-specific hot regions is near peri-telomere of chromosome 6, whereas female CO is almost absent. It is suggested that the mechanism of CO hot region determination may differ between sexes.

The intervals between two adjacent bins were defined as CO locations. And 92.6% (1216) and 81.1% (1506) of them could be limited to <200 kb in female and male, respectively, based on B73 v4 reference genome (Supplementary Figure 6). The higher resolution in female may partly be contributed by the higher sequencing coverage and several DNA copies (antipodal cells) for original MDA, while only single copy (single microspore DNA) was initiated in male. The 568 (43.3%) female and 350 (18.9%) male COs located under 10 kb were selected to analyze CO distribution at the gene scale. The relative distance from each CO (median position) to its closest gene was calculated (Fig. 2c). Comparing to five sets of randomly simulated positions, COs concentrate in the translation start sites and translation terminal sites of genes for each sex (Fig. 2c, two-sided Kolmogorov–Smirnov test *P* < 2.2 × 10^−16^ for female and male), which is consistent with the previous result based on the B73 v3 reference^26^. Intragenic enrichment of CO was also observed in yeast^33^ and in *Arabidopsis*^34^, but not in human^24,25^.

### Distinct CO interference patterns in different sexes

**Fig. 3:**
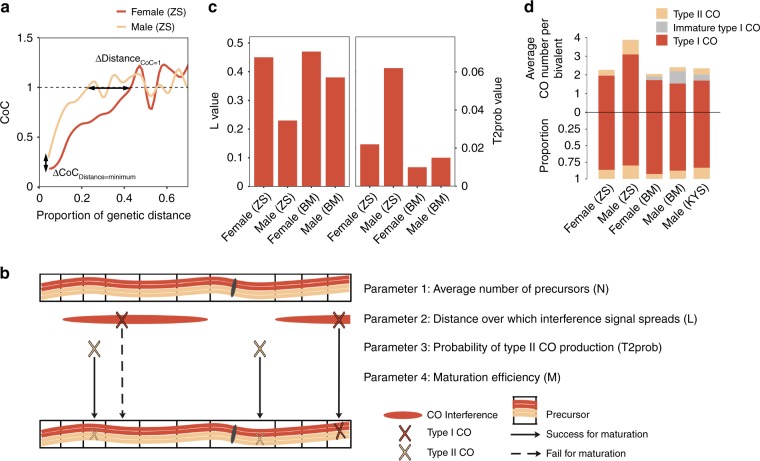
Features of crossover (CO) interference exhibiting sex difference. **a** The CoC curves for female and male gametophyte populations. Distance_CoC=1_ represents the distance at which CoC = 1. CoC_Distance=minimum_ represents the CoC value at minimum distance. ΔDistance_CoC=1_ and ΔCoC_distance=minimum_ represent the *D*-value of Distance_CoC=1_ and CoC_distance=minimum_ between sexes, respectively. **b** A diagram illustrating the Beam-Film model shows the main steps of CO formation and the main parameters for these steps. **c** The values of parameters *L* and T2prob in male and female meiosis within different genetic backgrounds. **d** The numbers (upper) and proportion (lower) of type I and II COs were shown. The putative numbers of immature type I COs derived from CMI were also present (gray in upper half of the graph). ZS is short for the F_1_ from the cross of inbred lines Zheng58 and SK, profiled by single gametophyte sequencing. BM is short for the F_1_ from the cross of inbred lines B73 and Mo17, profiled by sequencing progeny from reciprocal crosses. Source data of Fig. 3a are provided in a Source Data file.

CO interference was observed that COs inhibit the production of nearby COs^35–37^, preventing from CO clusters in each sample, thus increasing the evenness of CO distribution. In some organisms, such as yeast^38^, *Arabidopsis thaliana*^39^, maize^40^, and mouse^41^, most COs respond to interference, defined as class (type) I COs, produced via ZMM pathway^5^ (relying on Zip1, Zip2, Zip3, Zip4, Mer3, Msh4, and Msh5); whereas, the COs insensitive to interference are defined as class (type) II COs, dependent on Mus81 and Mms4/Eme1 pathway^37^. To profile CO interference in gametophyte populations, the coefficient of coincidence (CoC) was calculated that double CO rate in pair of intervals was divided by the product of two single CO rates in these intervals (see below), based on genetic distance. The CoC curve was plotted by the mean CoC for each distance between interval pairs. The difference in CoC curves suggests the sex-specific interference pattern and CO formation mechanism (Fig. 3a).

The fluctuant CoC values above 1 were found as a negative interference phenomenon in both sexes’ sets, which could be led from the positive interference as discussed in a previous study^43^. To accurately estimate interference, a mechanism-based CO simulation model, the BF model^42–44^ was used. This model was proposed to quantitatively explain experimental CO pattern datasets in many organisms, such as identifying the reduced interference in *top2* mutant of yeast^45^ and CMI in human female meiosis^46^, suggesting its reliability and robustness. In this model, a stress-and-stress relief mechanism was proposed to explain the formation of CO patterning (Fig. 3b). Briefly, a set of precursors are regularly distributed among and along bivalents. Mechanical stress promotes occurrence of CO designation during inter-homolog interactions. Precursors have varying stress response sensitivities. Average number of precursors was defined as parameter *N*. As stress is released, the precursor with the highest potential becomes interfering class I CO; then the local stress alleviates around this position, inhibiting the other precursors nearby. The distance that interference signal spreads was defined as parameter *L*, which reflects interference level. Independent of the process of class I CO designation, non-interfering class II CO could be generated from the precursors not becoming class I CO. The probability of class (type) II CO production was defined as parameter T2prob, which reflects the amount of class II COs. CMI would have happened, blocking some designated COs developing into actual COs. The mature rate of class I COs was defined as parameter *M* (Fig. 3b). These four main parameters (*N*, *L*, T2prob, and *M*) connecting biological process, critically determines CO pattern in meiosis (see all detailed parameters in Methods).

By determining values of the parameters (Supplementary Table 3), CO patterns could be simulated by the BF model with high repeatability. When simulated and observed CO sets share the fitting (1) CoC curve (along proportion of genetic distance), (2) average number and distribution of COs per bivalent, the parameter values could quantify the production of observed COs. When calculating CoC by using only the class I CO dataset, distance at which CoC = 0.5 (*L*_CoC=0.5_) in CoC pattern could evaluate interference level^43,46^; however, *L*_CoC=0.5_ is also affected by class II COs number (T2prob) when calculating CoC (Supplementary Figure 7a). Instead, we found that the distance at which CoC = 1 (Distance_CoC=1_) is invariable and the CoC value at minimum distance (CoC_Distance=minimum_) increases, when T2prob changes (Supplementary Figure 7b). These suggest that Distance_CoC=1_ and CoC_Distance=minimum_ directly reflect *L* and T2prob values, respectively. Through testing a range of parameter sets, the best-fit values for the two gametophyte populations within the Zheng58 × SK background were determined (*n* = 240 bivalents for male and *n* = 265 bivalents for female; Supplementary Figure 8). Varying parameters from the best simulation could not provide satisfactory match to the experimental data (Supplementary Figure 9), which indicated that these values of parameters are reliable.

Within the Zheng58 × SK background, higher Distance_CoC=1_ and lower CoC_Distance=minimum_ on female CoC curve (Fig. 3a) indicates a longer interference distance and less class II CO amounts in female meiosis, respectively. As determined by simulation, *L* in female (0.45) is 1.96 folds of *L* in male (0.23), while T2prob in female (0.022) is 0.35 folds of *L* in male (0.062) (Fig. 3c). To validate whether the sample sizes were enough to distinguish sex differences of *L* and T2prob, the bootstrapping analysis was performed by using another CO set observing late recombination nodules of microsporocyte of an inbred line KYS^14^ (*n* = 2080 bivalents). After randomly extracting 320 bivalents (in which CO number is equal to CO number in the embryo sac population) for 100 times, CoC curves of these sets were profiled (Supplementary Figure 10a). *D*-values of the Distance_CoC=1_ and CoC_distance=minimum_ in each pair of extracted sets were calculated (ΔDistance_CoC=1_ and ΔCoC_distance=minimum_). The *D*-values between female and male gametophyte sets within the Zheng58 × SK background were higher than 99.1% and 88.2% of the bootstrapped *D*-value sets, respectively (Supplementary Figure 10b), suggesting 99.1% and 88.2% confidence. The Zheng58 × SK COs from chromosomes with similar length were selected to simulate and to compare between sexes (Supplementary Figure 11). For long chromosomes (chr. 1–5, 135.71–108.19 μm for KYS male SC) and short chromosomes (chr. 6–10, 93.83–77.7 μm), *L* values were higher in female (0.30 and 0.46) than in male (0.19 and 0.27, respectively). We used higher *N* in long chromosomes (*N* = 18) than in short chromosomes (*N* = 12), as precursor number positively correlated with SC length. In this case, T2prob values of these sets in long and short chromosomes equal the values of the set in all chromosomes for both sexes (0.022 for female and 0.062 for male). These approved that the sex difference of parameters *L* and T2prob, independent of chromosome length. Moreover, based on the BF model, the best-fit parameters of CO sets from reciprocal crossing populations^23^ between F_1_ (B73 × Mo17) and B73 (*n* = 337.5 and 305 bivalents when F_1_ is taken as the maternal and paternal, respectively) were determined (Supplementary Figure 8). Compared with male meiosis, the higher *L* and the lower T2prob of female meiosis were also detected within the B73 × Mo17 background, which was similar with the Zheng58 × SK background (Fig. 3c; supplementary Table 3). Additionally, through simulating on a statistical oriented gamma model^40,47^ (Supplementary Figure 12), the stronger interference intensity (higher parameter *nu*) and less proportion of non-interfering COs (lower parameter *p*) in female meiosis was still detected within both backgrounds (Supplementary Figure 13). Combining these results, it could be concluded that the sex differences in interference strength and class II CO amounts are present, independent of backgrounds.

It’s puzzled that type I CO frequency within B73 × Mo17 background was higher in female (17.2 for BF and 18.2 for gamma model) than in male (15.4 for BF and 16.4 for gamma model), detected by using both BF (Fig. 3d) and gamma (Supplementary Figure 13) models; however, interference intensity is stronger in female meiosis than in male. These may only be explained by the unexpected CMI in this background, profiled by BF model. CMI was detected in male (*M* = 0.7) and female (*M* = 0.9) meiosis within the B73 × Mo17 background, as well as in KYS male meiosis (*M* = 0.84). For KYS CO set, at which *M* = 1, simulations always predict too many COs (2.5 COs for simulation and 2.0 COs for observation per bivalent, Supplementary Figure 14a) based on the best-fit CoC curve (Supplementary Figure 14b). In this case under *M* = 1, decreasing the number of active precursor by using highly skewed distribution of precursor sensitivity to CO designation (changing *A* from 1 to 6), decreased efficiency of precursor formation through DSB progress (changing *Y* from 1 to 0.5) and decreased average number of precursors per bivalent (changing *N* from 16 to 9) could reduce simulated COs to the experimental level (~2.0), but the simulated CoC curves would not be best fitted to the observed (Supplementary Figure 14). These three parameters were also not changed in human female meiosis with CMI^46^. For the CO sets within the B73 × Mo17 background, it was also proposed that M does not equal 1. Additionally, the bivalents without chiasmata were also observed in male meiosis of KYS^14^, recording with CMI. Combining these sets, CMI indeed exists in B73 × Mo17 and KYS, but not in Zheng58 × SK, suggesting that genetic background is probably key for CMI in maize.

Based on these parameters of BF model, it was calculated that both class I and II CO frequency of female (1.96 and 0.30 per bivalent) are less than that of male (3.10 and 0.78 per bivalent, respectively) within the Zheng58 × SK background (Fig. 3d). Fewer class I and II COs of female meiosis were also reported in *Arabidopsis*^48^. Although class I COs within the B73 × Mo17 background is more in female than in male meiosis based on BF and gamma models (see above), class II COs (0.14) and the designated class I COs (1.91), which are the sum of the mature and immature type I COs owing to CMI, are less in female than in male (0.22 and 2.19, respectively). These imply that COs of female meiosis are less than that of male unless CMI exists in maize. The precursor number (*N*) usually varies from 4 to 25 folds of chiasmata number in maize male meiosis^49–51^. We also tried to increase *N* (from 16 to 18, 20, 22, and 24) to simulate COs, resulting the increased CoC_Distance=minimum_ and the decreased T2prob value (from 0.062 to 0.048, 0.042, 0.038, and 0.034, respectively) (Supplementary Figure 15). However, the number and proportion of class I/class II COs were invariable within these pairs of parameters. Therefore, sex difference of class I and II number could still be concluded, no matter how *N* value changed.

## Discussion

We developed a method to sequence a single female gametophyte genome, through isolating antipodal cells from an embryo sac. This research suggests that sequencing single gametophyte genomes could be a general strategy to profile the sex-specific CO patterns of a single meiosis in other species as well. Analyzing meiotic CO is of fundamental importance for understanding the dynamics of genome evolution. Sex difference in CO pattern was reported in many species^10^. Here, under the same background of hermaphroditic species maize, sex differences in CO production could be accurately detected, owing to only caused by sex-specific activation of functional elements. Comparing the two datasets within the Zheng58 × SK background, it is surprising that many more COs were produced in male than in female (19.3 per microspore and 12.4 per embryo sac). This is consistent with the higher number of CO hot regions in male (112) than in female (71). Within the B73 × Mo17 background, although the CO numbers in male (8.6) meiosis accompanying CMI are slightly lower than in female (9.3), the sum of observed and immature COs derived from CMI is more in male (12.0) than in female (10.2). These suggest that COs in male may be more than in female unless CMI.

BF model is a comprehensive tool to explain the mechanism of CO production along longitudinal chromosome axis^42–44^. However, it is hard to capture synaptene nuclei in maize female meiosis, thus it is hard to detect female CO (chiasmata or MLH1) loci along SC. To compare the sex difference of CO interference, genetic distance was used to simulate for BF model, which could be reliable owing to that both SC length (μm) and genetic length (cM) of the chromosome are positively correlated with CO frequency. Although the different interference metrics (at genetic distance and SC length) may make various values of simulation parameters, the sex difference can still be revealed through comparing parameter values at the same interference metric (genetic distance). A much higher T2prob in male than female was found within the two backgrounds (Fig. 3c), illustrating that more class II COs formed during male meiosis (Fig. 3d). Since high temperature increases CO number through the class I pathway, but not class II pathway^52–54^, the detected sex difference of class II CO frequency could be independent of environment factors.

Based on the BF model, CMI was assumed as an underlying event during CO formation, which elevates aneuploidy in human female meiosis^46^. Therefore, dissecting the mechanism of CMI could be important for the understanding of CO production process and possible reducing instances of aneuploidy (like human Down’s syndrome). However, the molecular mechanism of CMI cannot be analyzed in human, since the functional elements of this female-specific process have yet to be fully understood. In maize, CMI may also contribute to pollen abortion and embryo aneuploidy. CMI was detected within the B73 × Mo17 and KYS backgrounds but not in Zheng58 × SK, suggesting that CMI may be triggered by a few background-specific elements (genes). Therefore, maize could be an ideal model to dissect the genetic foundation of CMI. With the development of a high-throughput method to detect CMI, the mechanism could be dissected further. Since CO number decreased with the presence of CMI (see Results), selecting the lines without CMI could increase the CO frequency thus enhancing the plant breeding efficiency. Moreover, introgression lines containing target regions without CMI could be used as males in backcross conversions, harboring more COs, especial class II COs, which would result in smaller recombination events in one generation.

The molecular mechanism of sex-specific CO production needs to be analyzed further. In this study, it was observed that SC length is positively related to CO number in both sexes. Nevertheless, nucleosome density, histone variant or modification (H2A.Z, H3K4me3, and H3K9me2), and DNA methylation level^55,56^ at synaptene were reported to influence DSB distribution and CO formation largely. Therefore, further analyzing the linkage between these molecular features and BF model parameters could deepen our knowledge of the mechanism of CO production.

## Methods

### Isolation of antipodal cells from single embryo sac

F_1_ individuals from a cross between Zheng58 and SK were planted in Wuhan, China. Ears with more than 15 cm silk were harvested and individual ovules were dissected out. The nucellus was isolated under stereoscope and mixed with a drop containing 1.5% pectinase (Sigma, Darmstadt, Germany), 0.5% pectolyase Y23 (Yakult, Tokyo, Japan), 1% cellulase RS (Yakult, Tokyo, Japan), 1% hemicellulase (Sigma, Darmstadt, Germany), and 0.54 M mannitol. Following 15–20 min incubation, the embryo sac was dissociated from the nucellus and was placed into a drop of 0.54 M mannitol by micropipette. A mass of antipodal cells was observed as cup structure at the chalazal end (Fig. 1), and could be cut off from embryo sac. Then, 5–15 antipodal cells were transferred into one PCR tube containing phosphate-buffered saline buffer from the REPLI-g Single Cell Kit (Qiagen, Hilden, Germany), and also can be stored at −80 °C for a long time for further use.

### Whole-genome amplification and sequencing

Based on the standard protocol of REPLI-g Single Cell Kit (Qiagen, Hilden, Germany), lysis of female gametophytes and amplification of whole genome (using MDA) was performed. To validate quality of MDA products, 10 markers differing between parents from 10 chromosomes (1 marker per 1 chromosome) were selected for PCR analysis (Supplementary Figure 1; see primers in Supplementary Table 4). The non-heterozygous markers should be present in haploid gametophytes. In total, 106 products, containing at least 8 qualified markers, were selected for library preparation using KAPA HyperPlus Library Preparation (Kapa Biosystems, Wilmington, MA). After sequencing on Illumina HiSeq 3000 platform in pair-end, 3.1 billion raw reads were obtained for next step of analysis.

### SNP calling and filtering

To obtain parental SNPs, sequencing data for parental inbred lines Zheng58^57^ and SK^26^ were downloaded. Reads were filtered by Trimmomatic^58^, then mapped to maize B73 AGPv4 genome^30^ by bwa^59^. The uniquely mapped reads were selected for calling SNPs, conducted by samtools^60^ and GATK^61^. Parental SNP filtration was according to the following criteria: (1) MAPQ ≥ 10; (2) SNPQ ≥ 30; (3) 3 ≤ DP ≤ 100 for SK and 4 ≤ DP ≤ 200 for Zheng58; (4) QD ≥ 2; (5) FS ≥ 20; (6) only homozygous SNPs were retained; and (7) distance between adjacent SNPs is more than 4 bp.

The sequencing data of embryo sacs and microspores sharing the same background^26^ were filtered by Trimmomatic^58^ and mapped to maize B73 AGPv4 genome^30^ by using bwa^59^. SNPs were called by samtools^60^, and filtered according to the following criteria: (1) homozygous SNPs; (2) SNPQ ≥ 20; (3) SNPs with minor allele frequency > 0.1; (4) SNPs can be detected in at least 10 samples; and (5) for each bin, at least 20 adjacent SNPs sharing the same parental background were required.

### Short bin validation

In this study, 20 short bins were identified totally (<1.5 Mb) in female gametophyte population. And we also re-mapped all the reads of male gametophyte population^26^ on the B73 v4 reference^30^ and there were 8 bins (all <1.5 Mb) identified in B73 v4 but not in B73 v3 reference. All the 28 bins were chosen for further validation since double COs in small regions are rare^7,31^. Based on sanger sequencing, it would be validated if the parental haplotypes and progenies haplotypes are as expected from Illumina sequencing. However, considering the complex maize genome, not all the designed primers can be amplified and sequenced successfully in both parents and progenies. Finally, 9 of 20 female short bins and 3 of 8 male short bins were validated as positive; 3 of 20 female short bins and 1 of 8 male short bins were validated as false positive; and others cannot be validated as positive or false positive due to technical issue. The invalidated and false positive short bins have been eliminated for further analysis. For each bin, the detail results and primer sequences could be found in Supplementary Tables 2 and 4.

### CO simulation by using BF model

The BF model mathematically simulates the biological processes of CO production. Parameters in this model could be divided into three classes^46^. The first class determines the array of active precursors, including: (1) *N*, average number of precursors per bivalent; (2) *E*, precursor spacing along bivalent; (3) *B*, precursor distribution in population; (4) *Y*, efficiency of active precursor formation from DSB; and (5) *A*, distribution of sensitivity among different precursors. The second class describes the patterning of (interfering) CO designation, including: (1) Smax, starting stress level relative to sensitives of precursors; and (2) *L*, distance over which interference signal spreads. The third class describes the post-patterning process, including: (1) *M*, probability of that CO-designated interactions will successfully develop to mature COs; and (2) T2prob, probability of that CO-failed precursors will eventually progress to type II COs.

The SC length is not available in female of maize, but the genetic distance determined by using QTL IciMapping^62^ was used for CoC curve fitting, owing to that both SC and genetic distance are positively correlated with CO number. In the CoC curves of both sexes against absolute genetic distance (Supplementary Figure 16), similar Distance_CoC=1_ values were observed, which is consisting with the result in the curves against SC length^25^. Through determining the values of the parameters by many times of trial and error, best-fit predictions can be generally approached. However, the simulated COs are at the bivalent level, while the experimental is at the chromatid level. Therefore, CO array exported from BF model simulation needs to be transformed to the chromatid level. In detail, one CO in bivalent could happen in four pairs between each two chromatids, with the same probability (25% for each pair) (Supplementary Figure 17a); consequently, these pairs for all COs in whole bivalent were randomly combined to generate CO sets at the chromatid level, based on binomial distribution. The simulated (bivalent level) and transformed (chromatid level) CO sets, as well as the observed CO sets of tetrad and microspore level, share the same Distance_CoC=1_ value and CoC_Distance=minimum_ value (Supplementary Figure 17b). Owing to the large sample size (*n* = 5000), the CoC curves of simulated and transformed sets almost coincide. In addition, the average number and distribution of CO in simulated (bivalent level) and transformed (chromatid level) sets correspond to those in tetrad (bivalent level) and microspore (chromatid level) set, respectively (Supplementary Figure 17c). These suggest that the transformation is reliable and accurate.

### CO simulation by using gamma model

Gamma model mathematically described the distribution of inter-CO distance, estimating interference strength using parameter *nu*. Parameter *p* was defined as the proportion of non-interfering COs^40,63^. Simulating inter-CO distance distribution using gamma model was conducted by CO distribution analyzer (CODA) software^64^ (version 1.1), following the manual. We set simulation number at 5000 and used maximum likelihood method to obtain the best-fit inter-CO distance distributions.

### Code availability

BF model analysis was conducted by previously published MATLAB program, which is available at https://projects.iq.harvard.edu/kleckner_lab/lab-software.

### Reporting summary

Further information on experimental design is available in the Nature Research Reporting Summary linked to this article.

## Supplementary information

Supplementary Informations

Description of Additional Supplementary Data

Supplementary Data 1

Supplementary Data 2

Supplementary Data 3

Supplementary Data 4

Reporting Summary

Source Data File

## Data Availability

Data supporting the findings of this work are available within the paper and its Supplementary Information files and from the corresponding authors upon reasonable request. The sequencing dataset for single gametophyte genomes were deposited in the NCBI BioProject under accession code PRJNA489007. The source data underlying Fig. 3a and Supplementary Figures 1, 7, 8, 9, 10, 11, 15a, 16, and 17 are provided as a Source Data file.
