# Supplementary material for: Single gametophyte sequencing reveals that crossover events differ between sexes in maize
Source: Nat Commun. 2019 Feb 15;10:785. doi: 10.1038/s41467-019-08786-x (PMC6377631; doi:10.1038/s41467-019-08786-x)
Supplement: Supplementary file 1 — Supplementary Informations [file 41467_2019_8786_MOESM1_ESM.pdf]

Single gametophyte sequencing reveals that crossover events differ between sexes in maize

Luo *et al.*

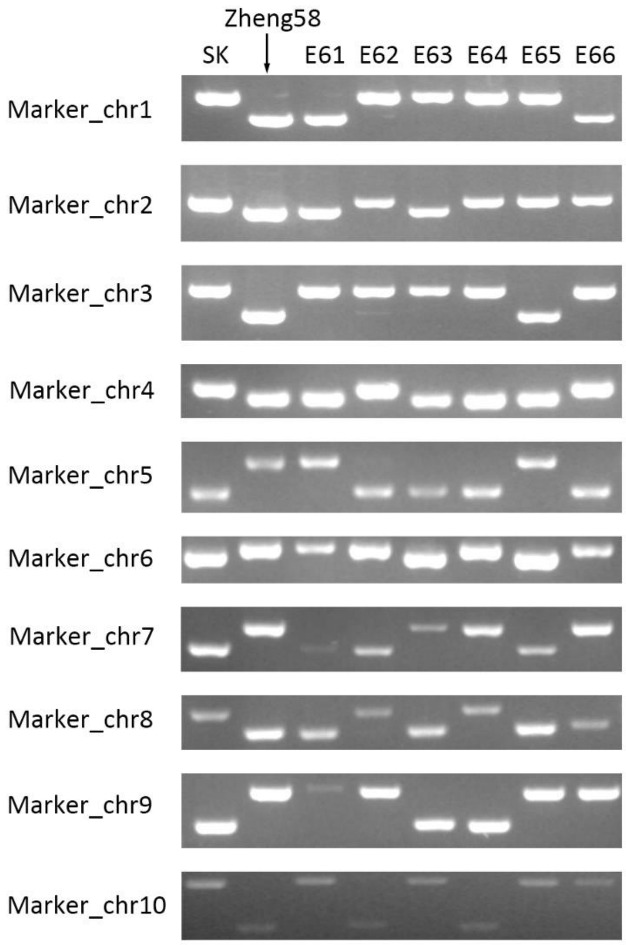

Supplementary Figure 1. The examples of MDA products detected by molecular markers. Per each chromosome, one polymorphic marker between parents was selected to detect all amplified products. The first two columns show parental polymorphisms of these makers. The other six columns show the single gametophyte samples covered well with ten non-heterozygous markers, which were qualified to make library and to sequence. Source data are provided in a Source Data file.

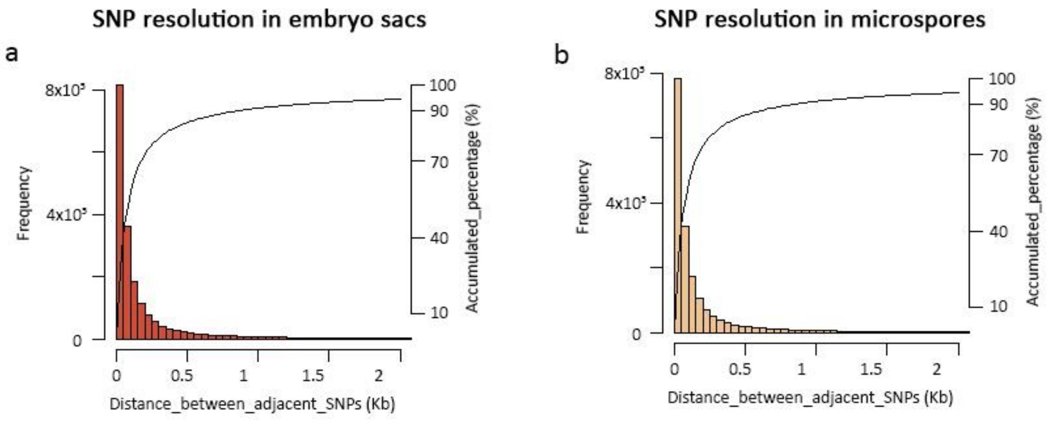

Supplementary Figure 2. SNP resolution of the sequencing dataset. The histogram and accumulated percentage of adjacent SNPs' distances were illustrated for embryo sac (a) and microspore (b) sets. The reads from single microspores were previously sequenced. In the research, they were re-aligned at B74 AGPv4 reference. After calling and filtering SNPs with the same standard as the single embryo dataset, the high-quality SNPs were obtained.

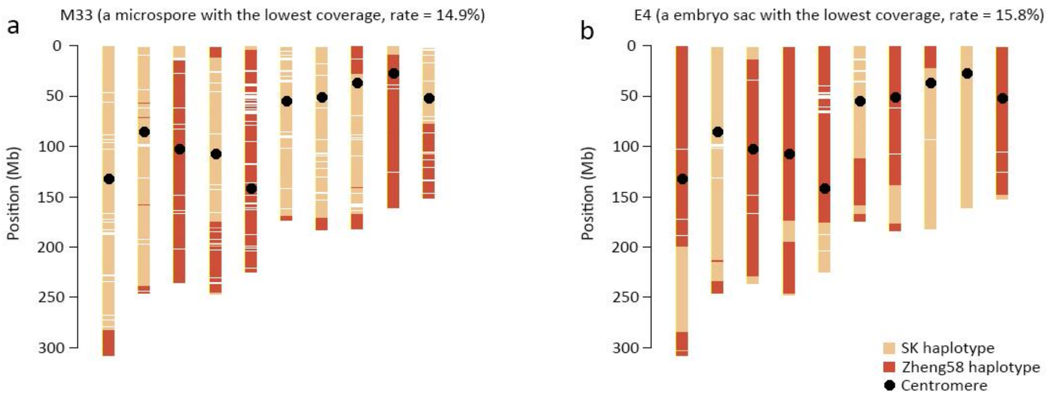

Supplementary Figure 3. The bin maps of the microspore and embryo sac samples with the lowest genome coverage. Each horizontal line represents parental SNP. After filtering, SNPs of M33 (a microspore with the lowest coverage, 14.9%, with 485K SNPs) and E4 (an embryo sac with the lowest coverage, 15.8%, with 520Ks) were distributed evenly along chromosomes.

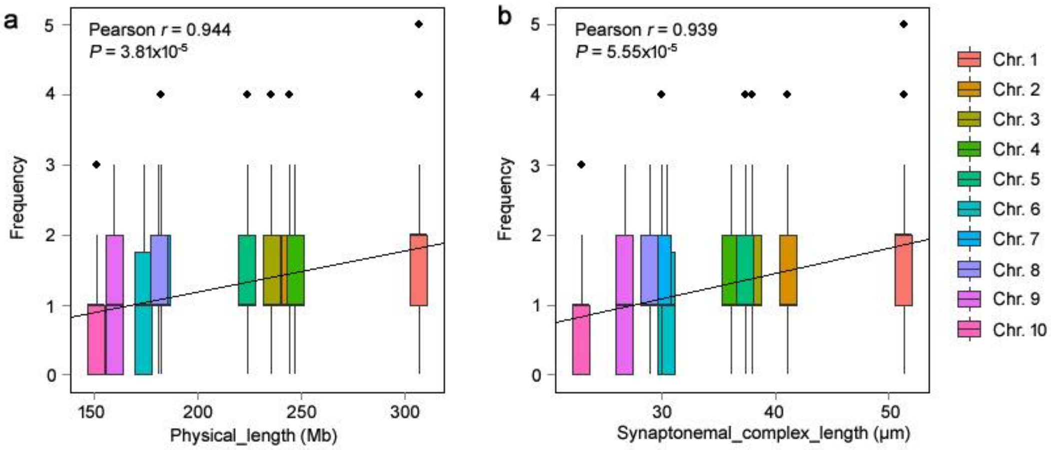

Supplementary Figure 4. CO amount per chromosome is correlated to chromosome length. Both the physical length (a) and SC length (b) of chromosome are significantly correlated to CO amount (Pearson *r* = 0.944 and 0.939; *P* = 3.81 x 10^{-5} and 5.55 x 10^{-5}, respectively). SC length was detected in inbred line KYS (Falque et al., 2009). In each box, the bold horizontal line represents the median and vertical lines mark the range from 5th and 95th percentile of the total data.

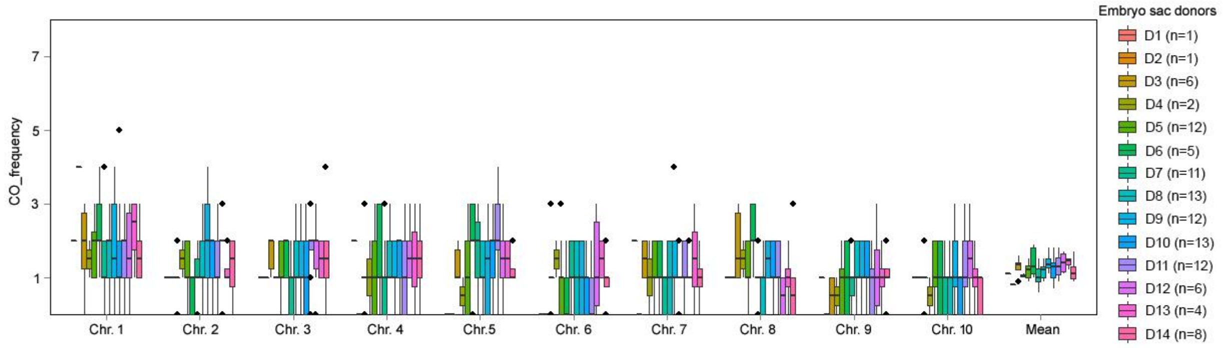

Supplementary Figure 5. CO frequency of single embryo sacs does not vary in donors. The 106 embryo sacs were isolated from 14 individuals (donors). No significant difference of CO frequency among donors was uncovered. In each box, the bold horizontal line represents the median and vertical lines mark the range from 5th and 95th percentile of the total data.

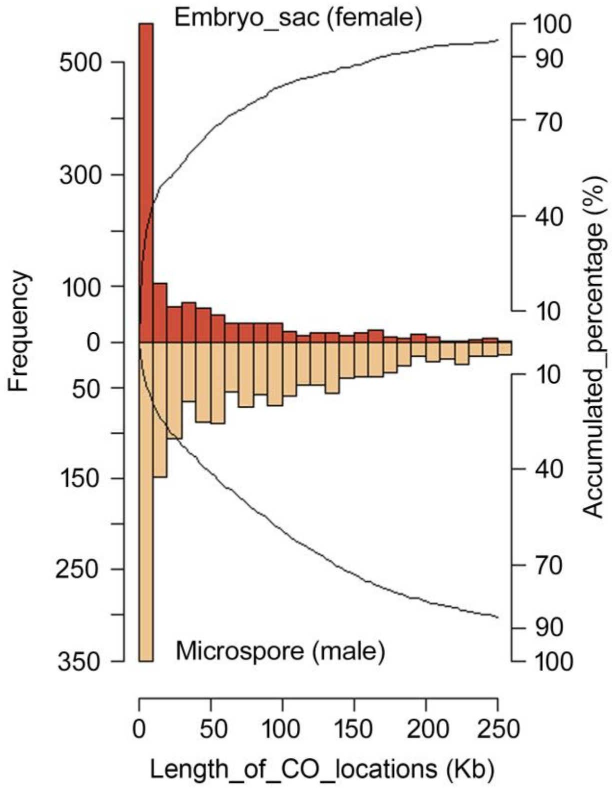

Supplementary Figure 6. The limited length of CO locations detected in female and male. The high numbers of locations (intervals) with limited length illustrate a high accuracy of SNP calling and CO identification.

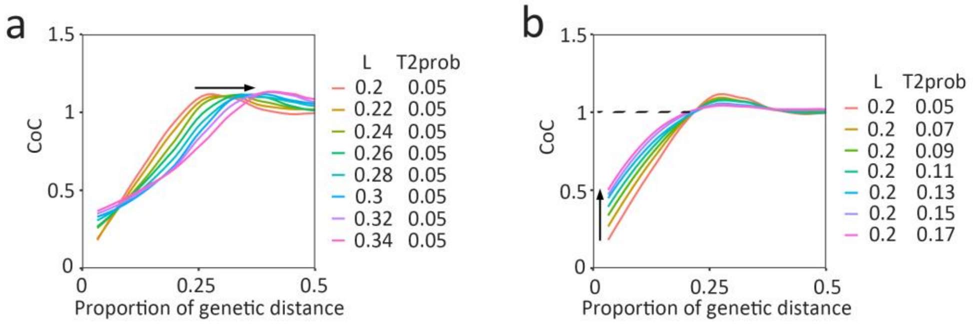

Supplementary Figure 7. The principle to determine L and T2prob value in Beam-Film model. (a) When T2prob value is fixed, the interval $\text{Distance}_{\text{CoC=1}}$ is growing as only L value is increasing. (b) When L value is fixed, the $\text{CoC}_{\text{Distance=minimum}}$ is growing as only T2prob value is increasing, but the interval $\text{Distance}_{\text{CoC=1}}$ is constant. These suggest that the interval $\text{Distance}_{\text{CoC=1}}$ and $\text{CoC}_{\text{Distance=minimum}}$ are key factors to determine L and T2prob value. Source data are provided in a Source Data file.

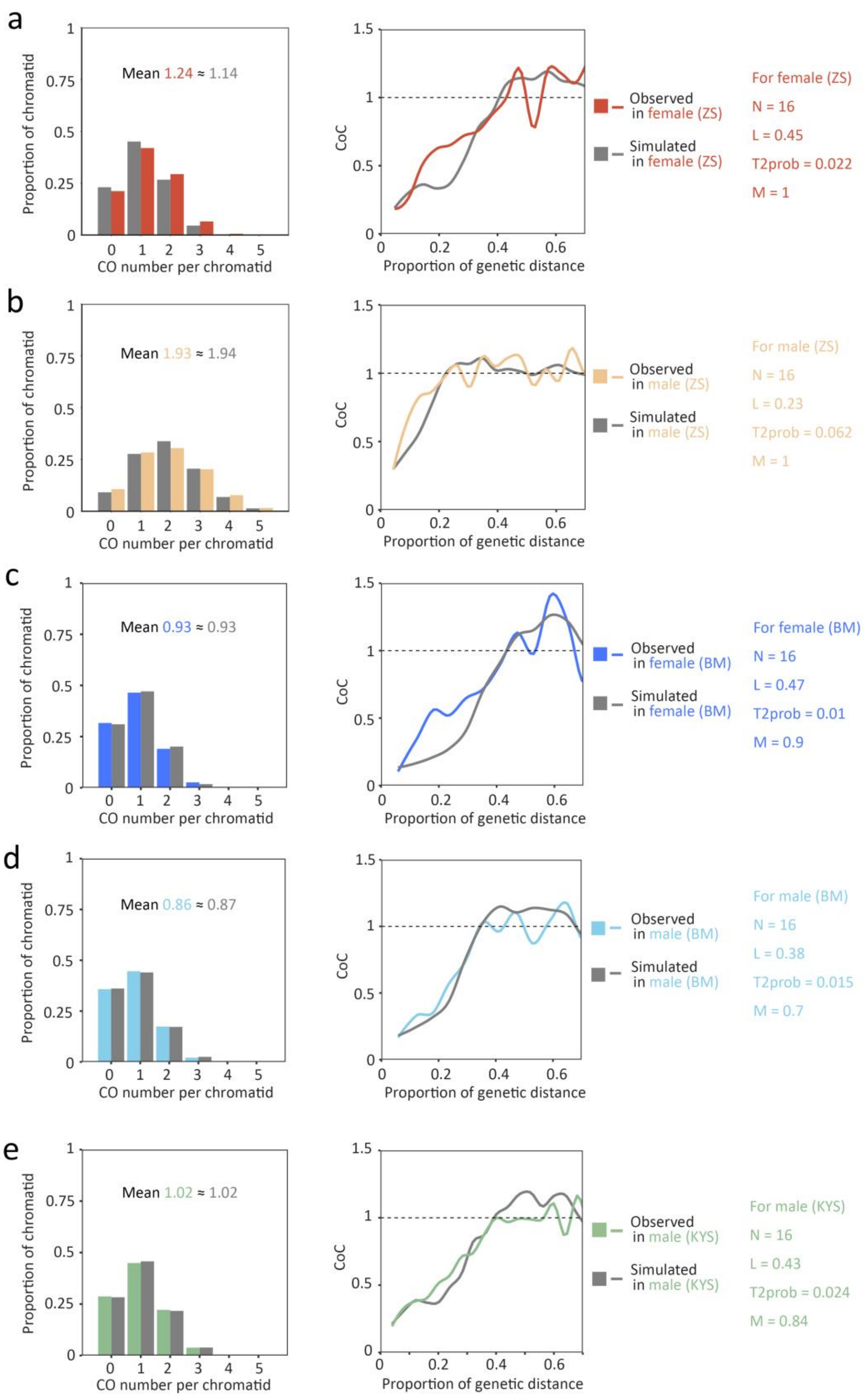

Supplementary Figure 8. The determination of the major parameters. The CO number per chromatid and CoC patterns were estimated by using whole genome COs for

five datasets: (a) female (ZS), (b) male (ZS), (c) female (BM), (d) male (BM), and (e) male (Inbred). Through modifying value of parameters, the simulated samples were produced. Because the best fit simulation shares the same CO number per chromatid and CoC pattern with observed data, the value of parameters for simulation could also represent the features of observed COs. The major parameters (N, L, T2prob, and M) were shown for each set. ZS is short for the F_1 crossing from the regular lines Zheng58 and SK, profiled by single gametophyte sequencing. BM is short for the F_1 crossing from the regular lines B73 and Mo17, profiled by sequencing progeny from reciprocal cross. See the detail parameter list in Supplementary Table 3. Source data are provided in a Source Data file.

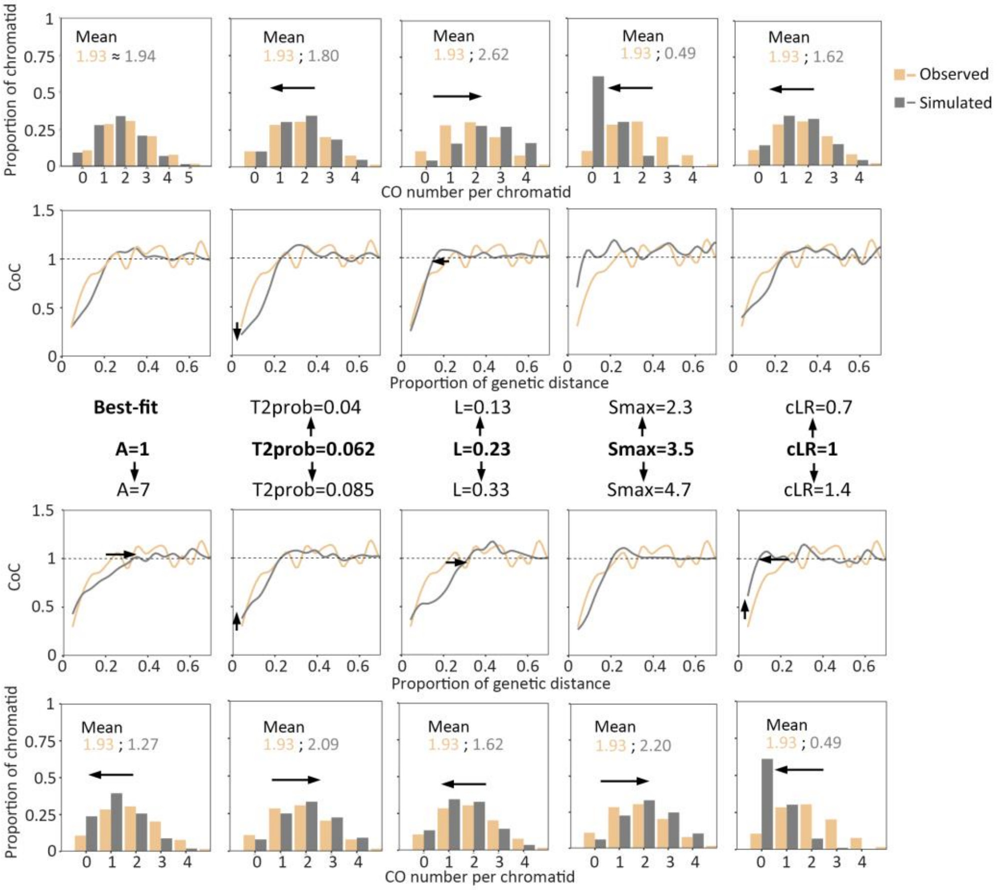

Supplementary Figure 9. The accuracy of value for major parameters. For the male population within Zheng58 x SK background, the CO number per chromatid or/and CoC pattern would be changed, as the value of major parameters (including A, T2prob, L, Smax, and cLR) are changing. It suggests that the value of these parameters may be unique for best fit simulation. Source data are provided in a Source Data file.

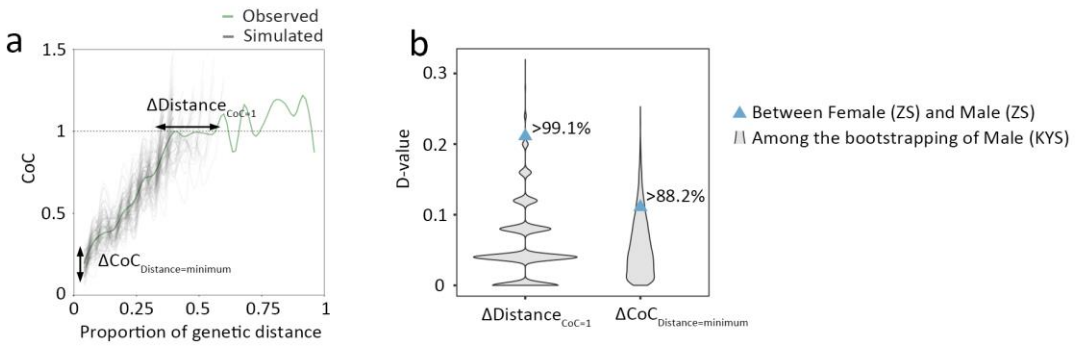

Supplementary Figure 10. Sample size is enough for single gametophyte populations. (a) 320 bivalents were randomly selected from total 2080 bivalents of inbred line for 100 times. The CoC pattern (only for CoC ≤ 1) of these selected datasets were shown as light grey lines. (b) the Distance (at which CoC=1) and the CoC (at which Distance is minimum) of these bootstrapping sets were obtained. The violin plots of D-values among them were illustrated. Additionally, the D-values between microspore and embryo sac populations were also highlighted as blue triangles, which are more than 99.1% of $\Delta Distance_{CoC=1}$ and 88.2 % of $\Delta CoC_{Distance=minimum}$ values from the bootstrapping sets. ZS is short for the F_1 crossing from the regular lines Zheng58 and SK. Source data are provided in a Source Data file.

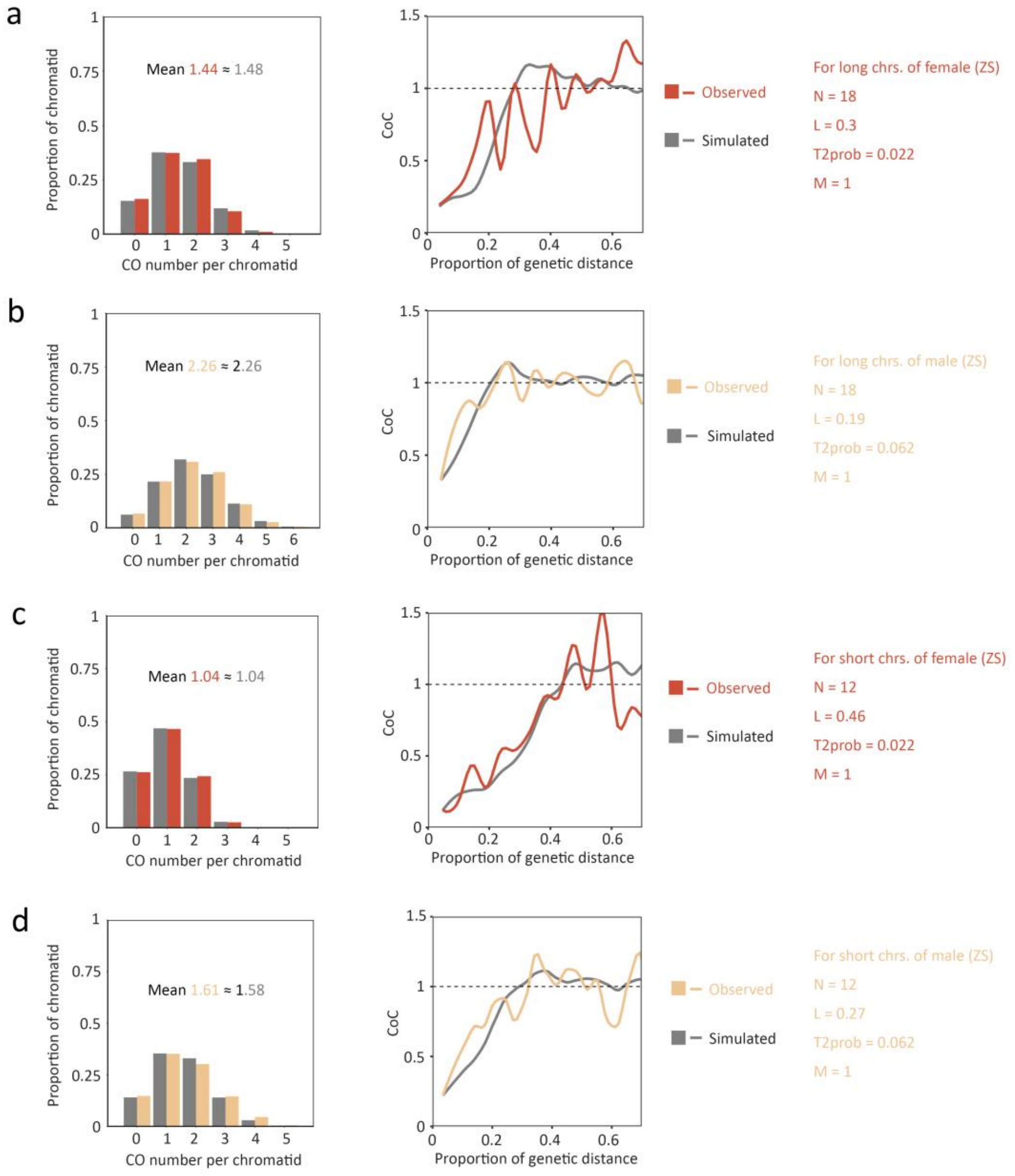

Supplementary Figure 11. The best-fit simulations of CO patterns from chromosomes with similar length. In the Zheng58 x SK background, COs from (a) long chromosomes of female, (b) long chromosomes of male, (c) short chromosomes of female, and (d) short chromosomes of male were simulated. Chromosomes 1 to 5 were defined as long chromosomes, and chromosomes 6 to 10 were defined as short chromosomes. ZS is short for the F_1 from the cross of inbred lines Zheng58 and SK. Source data are provided in a Source Data file.

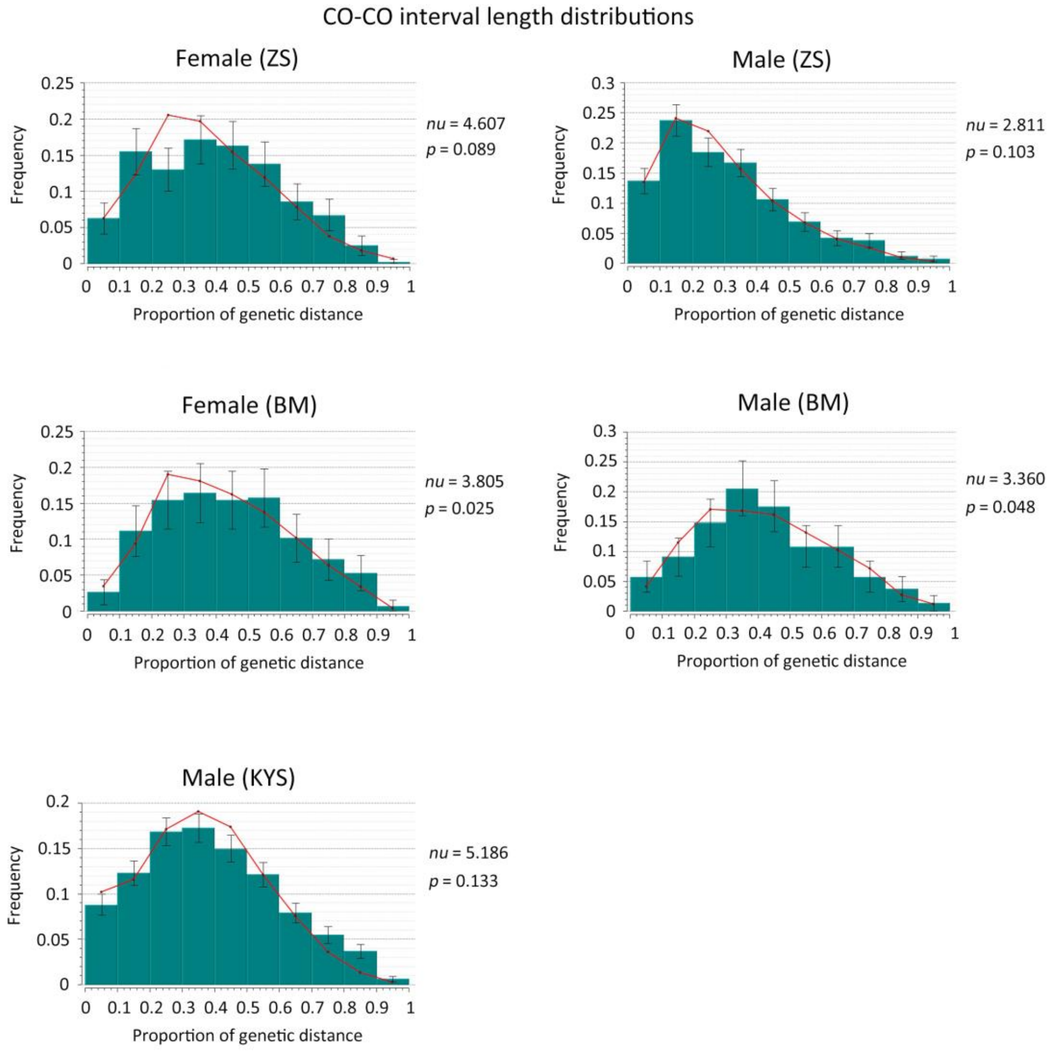

Supplementary Figure 12. The best fit simulation based on gamma model. For each population, the inter-interval lengths of simulated (red line) and observed (green box; bar represents the range from 5th and 95th percentile) COs were distributed, and best fitting.

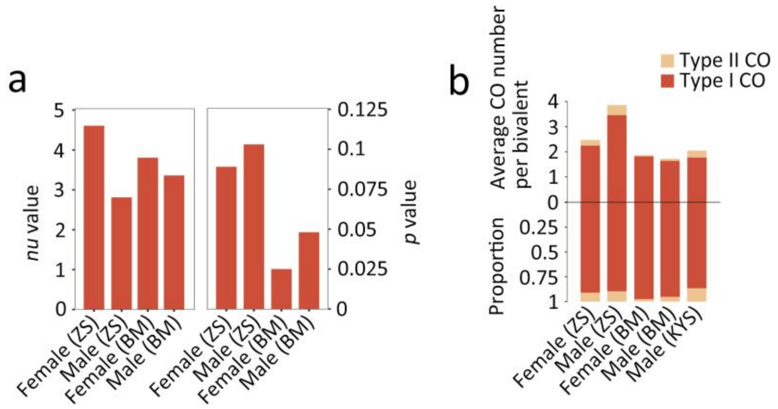

Supplementary Figure 13. Numbers of interfering and non-interfering COs estimated by gamma model. (a) The parameters, including interference intensity (*nu*) and proportion of non-interfering COs (*p*) were shown for each population. (b) The average number per bivalent and the proportion of types I and II COs, estimated by gamma model, were shown.

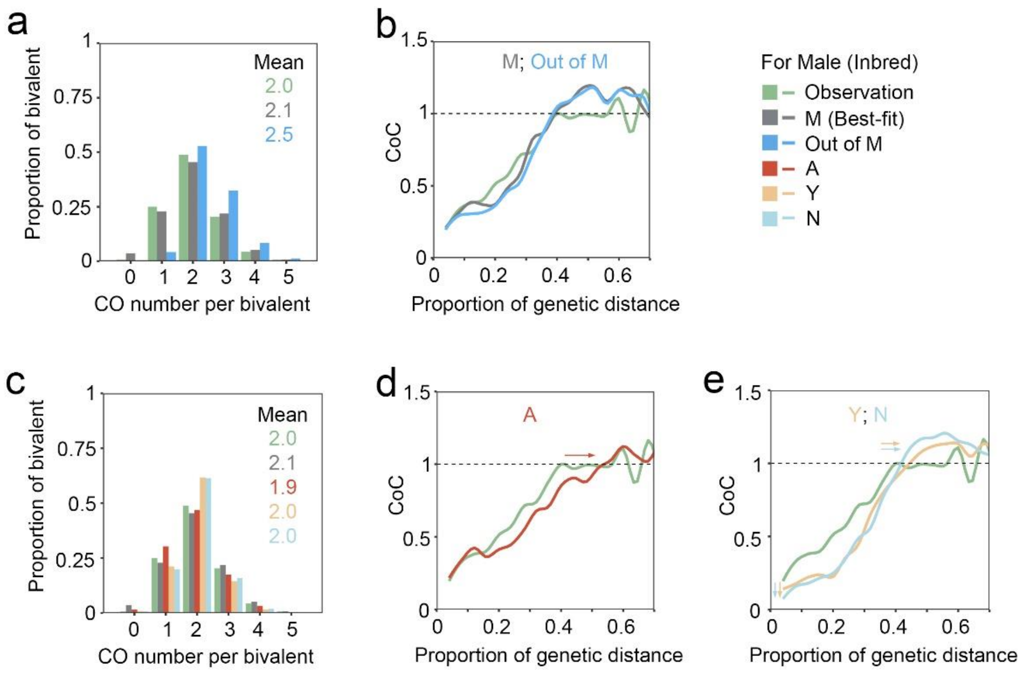

Supplementary Figure 14. CMI (M<1) is necessary for male CO production in KYS. The distributions of CO number per bivalent (a) and CoC patterns within different CO distances (b) were shown for Observation, “M” and “Out of M” datasets. Although both “M” and “Out of M” share the same CoC pattern with observation (b), the mean of CO numbers per bivalent for “Out of M” (2.5) are much higher than them for observation (2.0) and “M” (2.1) datasets (a). The CO frequency mean can be reduced through compensating to change A, Y, and N value (c). However, Changing A value could move the $\mathrm{Distance}_{\mathrm{CoC=1}}$ (d), and changing Y and N value could move the $\mathrm{Distance}_{\mathrm{CoC=1}}$ and $\mathrm{CoC}_{\mathrm{Distance=minimum}}$ (e). These suggest that M=0.8 is necessary for simulation of the inbred line dataset. In each panel, “M” is the abbreviate for simulation considering M value as 0.8, which is best fit (M=0.8, A=1, Y=1, N=16) for observation. “Out of M” is the abbreviate for simulation with complete M value (M=1, A=1, Y=1, N=16). “A” is the abbreviate for simulation changing A value (M=1, A=6, Y=1, N=16). “Y” is the abbreviate for simulation changing Y value (M=1, A=1, Y=0.5, N=16). “N” is the abbreviate for simulation changing N value (M=1, A=1, Y=1, N=9).

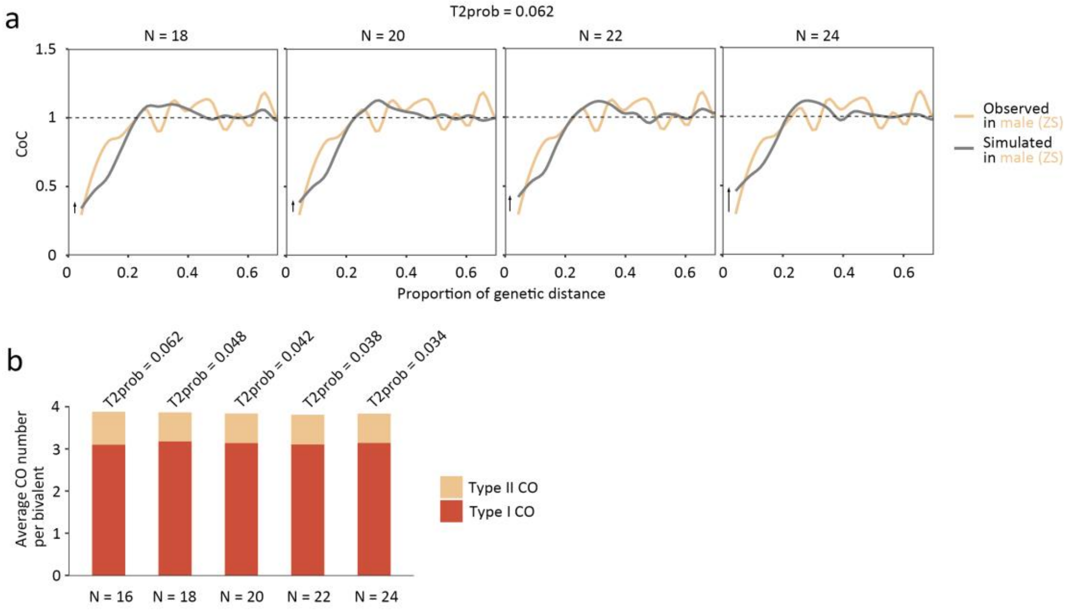

Supplementary Figure 15. Ratio of Type I/II COs is invariable when Increasing N value and decreasing T2prob value. (a) CoC curves of simulated sets when increasing N value and freeze T2prob value. The $CoC_{Distance=minimum}$ values rise. (b) For the best-fit simulated sets with higher N and lower T2prob, the numbers and ratio of Type II and I COs are relatively invariable. Source data of Supplementary Figure 15a are provided in a Source Data file.

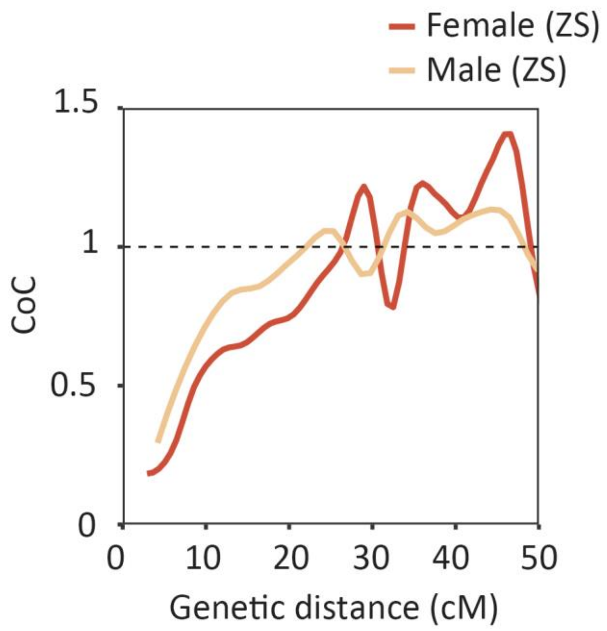

Supplementary Figure 16. The CoC curves for female and male gametophyte populations (ZS) against the absolute genetic distance. ZS is short for the F_1 from the cross of inbred lines Zheng58 and SK, profiled by single gametophyte sequencing. Source data are provided in a Source Data file.

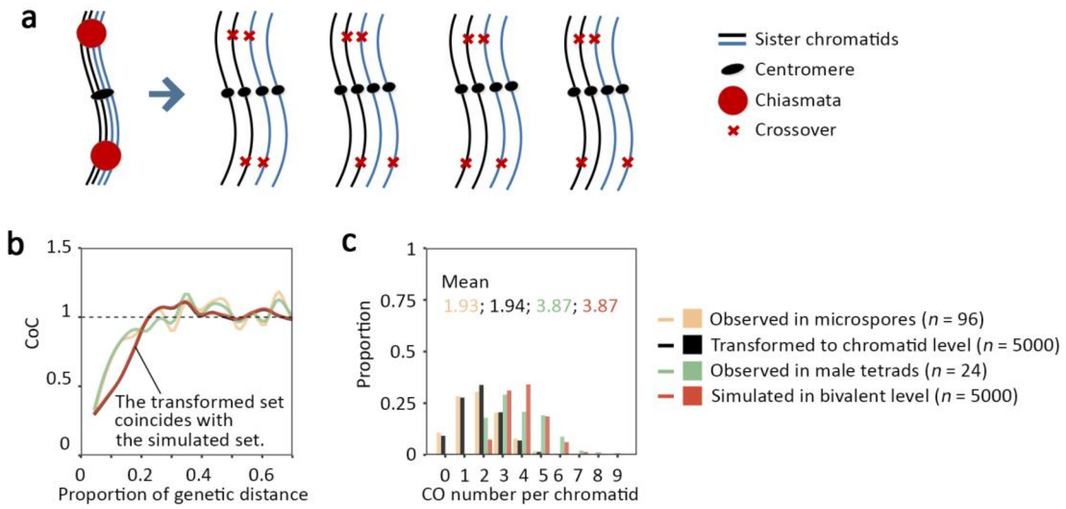

Supplementary Figure 17. Transformation from CO dataset on bivalent to on chromatid. (a) The process of transformation. The CoC patterns (b) and the CO frequency (c) of these four sets were illustrated, including the observed COs in 1) microspore and 2) tetrad levels under the Zheng58 x SK background, 3) the simulated COs in bivalent level, and 4) the transformed COs in chromatid level. Source data are provided in a Source Data file.

Supplementary Table 1. SNP count of 10 chromosomes in female and male ametophyte populations*

Chr	Raw SNPs	High-quality SNPs (female)	High-quality SNPs (male)
**chr1**	561,149	283,595	271,170
**chr2**	426,204	225,436	212,763
**chr3**	460,169	249,319	232,837
**chr4**	483,650	271,325	241,249
**chr5**	367,306	179,825	169,281
**chr6**	296,754	159,744	149,737
**chr7**	326,919	181,506	160,679
**chr8**	326,710	183,529	169,844
**chr9**	293,589	158,192	151,466
**chr10**	257,060	140,945	132,511
**total**	3,799,510	2,033,416	1,891,537

* Zheng58 x SK background

Supplementary Table 2. Validation of short bins in male and female gametophytes by sanger sequencing*

ID	Chr	Sample	Pos_start (bp)	Pos_end (bp)	Length	Validation**
F-1	1	E3	297,613,159	298,520,965	907,806	Validated (11 SNPs)
F-2	1	E12, E93, E109	302,864,469	303,247,472	383,003	Validated (1 SNP)
F-3	1	E69	283,671,920	283,960,204	288,284	Validated (9 SNPs, 3 INDELs)
F-4	2	E50, E54, E60, E70, E92, E93, E95, E96	213,989,550	214,206,806	217,256	Failed
F-5	2	E60	139,688,284	140,012,686	324,402	Failed
F-6	2	E69	142,000,138	142,272,836	272,698	Validated (29 SNPs, 7 INDELs)
F-7	2	E74	240,607,189	241,031,741	424,552	Validated (6 SNPs, 1 INDEL)
F-8	2	E74	241,033,291	241,699,631	666,340	Validated (3 SNPs)
F-9	2	E86	232,006,411	232,339,093	332,682	False
F-10	3	E108	230,192,560	230,397,263	204,703	Validated (2 INDELs)
F-11	4	E56, E61, E62, E64, E80, E81, E83, E85, E86	137,737,575	138,201,909	464,334	False
F-12	5	E57	191,983,161	192,196,143	212,982	Failed
F-13	5	E82	173,746,853	174,145,321	398,468	Failed
F-14	5	E82	174,205,749	174,740,720	534,971	Failed
F-15	5	E88	220,044,645	220,945,789	901,144	Validated (5 SNPs, 1 INDEL)
F-16	6	E49	60,461,981	61,008,657	546,676	Failed
F-17	7	E8, E32, E91	178,741,467	179,171,204	429,737	Validated (2 SNPs)
F-18	7	E20, E54, E93	177,282,243	177,701,384	419,141	Failed
F-19	7	E69	89,948,324	90,489,377	541,053	False
F-20	8	E40	166,376,903	167,552,857	1,175,954	Failed
M-1	1	M37, M39	247,686,168	248,215,429	529,261	Validated (3 SNPs)
M-2	1	M37, M39	268,828,095	268,979,065	150,970	Failed
M-3	1	M89, M92	35,366,399	35,817,198	450,799	False
M-5	2	M5, M7, M91, M92	241,387,860	241,699,631	311,771	Failed
M-6	2	M7, M91, M92	240,607,189	241,312,811	705,622	Validated (6 SNPs)
M-7	3	M17, M20, M29, M31, M53, M56, M66, M67, M89, M90	20,591	863,477	842,886	Failed
M-8	3	M37	217,573,788	218,481,150	907,362	Validated (3 SNPs)
M-10	7	M33	18,229,587	18,718,847	489,260	Failed

* Zheng58 x SK background.

** Including three conditions. “Validated” means successful validation, “False” means non-successful validation, “Failed” means unsuccessful genotyping due to technique issue.

Supplementary Table 3. Simulation parameters used in BF model

Set	N	B	E	Bs	Be	Bd	Y	Smax	Bsmax	A	L	cL	cR	M	T2prob
Female-ZS	16	0.6	0.6	1	1	1	1	3.5	0.9	1	0.45	1	1	1	0.022
Male-ZS	16	0.6	0.6	1	1	1	1	3.5	0.9	1	0.23	1	1	1	0.062
Female-ZS (for long chrs.)	18	0.6	0.6	1	1	1	1	3.5	0.9	1	0.30	1	1	1	0.022
Male-ZS (for long chrs.)	18	0.6	0.6	1	1	1	1	3.5	0.9	1	0.19	1	1	1	0.062
Female-ZS (for short chrs.)	12	0.6	0.6	1	1	1	1	3.5	0.9	1	0.46	1	1	1	0.022
Male-ZS (for short chrs.)	12	0.6	0.6	1	1	1	1	3.5	0.9	1	0.27	1	1	1	0.062
Female-BM	16	0.6	0.6	1	1	1	1	3.5	0.9	1	0.47	1	1	0.9	0.01
Male-BM	16	0.6	0.6	1	1	1	1	3.5	0.9	1	0.38	1	1	0.7	0.015
Male-KYS	16	0.6	0.6	1	1	1	1	3.5	0.9	1	0.43	1	1	0.84	0.024

Supplementary Table 4. Primers used in this study

Name	Sequence	Note
marker_chr1	5'-AAGTGGTGAGGTAAGCCTGC-3'/5'-ATAGGAGACACCCTGGGCAT-3	Detection of amplification coverage before making libraries.
marker_chr2	5'-CTCTTCCAATCGGGTTTGC-3'/5'-AATTGCACATAACAGAGGCG-3'	
marker_chr3	5'-CTCAGGAGGAGGAAATGTGG-3'/5'-CTTCTGTCCGTGAAGGATGG-3'	
marker_chr4	5'-AAGAACAGCATTGTCGTCACC-3'/5'-GTCCAGCGTCAGAGCTTACC-3'	
marker_chr5	5'-AAAGCACTTACATCATGGGAAAC-3'/5'-TTGGTGTAGCTCCGATTTG-3'	
marker_chr6	5'-CATAGTCCGATCTTGGTGACG-3'/5'-CATACAGGGAGTCACGGTCC-3'	
marker_chr7	5'-AGCACCAGGAAGTTGTGAGG-3'/5'-CCAACTCGATACGAAGAGCC-3'	
marker_chr8	5'-TCCAAGTCCCATGGCAGAAC-3'/5'-CCATGCTAGCTGATCGTCGT-3	
marker_chr9	5'-CCCGAGTTGCATGGAAGACA-3'/5'-CGTGCGTTTGAATTGGCTGA-3'	
marker_chr10	5'-CCTTCTAATTAAAGTCAAAGCCA-3'/5'-CAACACCCAACATCCGTGCT-3'	
F-1	5'-CTTGTCCAACTAGCCTCAACAC-3'/5'-ACGCCAAATCTTGACCAGCAT-3'	Validation of short bins in female gametophytes (Zheng58 x SK background)
F-2	5'-CCACGCAAAGTCCAAAACCC-3'/5'-TCCTTCCAGGTGCAGTAGGT-3'	
F-3	5'-CAAGTCAACAAGGCGCGAAA-3'/5'-GACATGGACGAGAGGGAGGA-3'	
F-4	5'-CAGCCGAGATCCCAGACAAA-3'/5'-CAACACCTAGACGGTTGACGA-3'	
F-5	5'-TGCGTCCTCACTCCTCAATC-3'/5'-GCTGTCTTCTCCTCATCGGG-3'	
F-6	5'-AGTCATCTATCCCGATCAACCCC-3'/5'-GACGGCTCAGTGTGTTCTCAT-3'	
F-7	5'-GTCAACAAAGAGGACGGCAC-3'/5'-GGCTTGCTGTCTTCTCCCAT-3'	
F-8	5'-ACCAAGAGATCGAAGGTCACAA-3'/5'-TCCAAGCGTATAGCAAAGGAAG-3'	
F-9	5'-GCATTCTCAAGGTCGCATGG-3'/5'-TCGGGCTTCGTTTCAGTCTT-3'	
F-10	5'-TGTCGCTAGCCGTAGGTATG-3'/5'-TGGCAGAGGCATCAGTATCAC-3'	
F-11	5'-AGTACGTGATAGCGACCTGC-3'/5'-CCAGCAGAAAGCATGAGGGA-3'	
F-12	5'-CCAACTCAGGGCGGTCATC-3'/5'-ATCCACGTTTCTGGTCTCTGAAC-3'	
F-13	5'-CACCAGCTTCCTGTCGAGTT-3'/5'-TCAGCCCAAGAAGAGGAACG-3'	
F-14	5'-TGCGAGAGTGAAAGAGGCAA-3'/5'-GTGCTGACAGTCTTCTGGAGT-3'	
F-15	5'-ATCTTGGGTGATGATGTCGAGC-3'/5'-GCTGAGTGCTACAAAATTCTCGG-3'	
F-16	5'-AGGGTTCCCCATCTCTGTGT-3'/5'-TGCTCGTCCTTGATGCCAAA-3'	
F-17	5'-GCGAGTTCCAGTGCTACGA-3'/5'-AACTCCATGAACTGCGGGAA-3'	
F-18	5'-AGTGTTGGCAGTGTTTCCTCATAC-3'/5'-TCATTGGACAGGCGCTAGA-3'	
F-19	5'-CCTCTTTCTTGTCGCACCCA-3'/5'-GCTGATGTCCTTGCTCACCT-3'	
F-20	5'-TTGACCCACACGACCAGAAC-3'/5'-CATGATCTCGTCGGCCTTCA-3'	
M-1	5'-TTTTCCTGGGGTTAGGCGAAT-3'/5'-TGTTGTCCATGTTGCGTCATT-3'	Validation of short bins in male gametophytes (Zheng58 x SK background)
M-2	5'-GCGGCTACTGTTGAGGTAGA-3'/5'-GGCTGGGGTGCTCTGTATTT-3'	
M-3	5'-AAGAAGTACCCCGACGCAAG-3'/5'-TGTTTCTGCCCGTGCTGG-3'	
M-5	5'-ATCTCCTTCTTCAGGCGGTG-3'/5'-CGAAGGAAGCCATCTCGTCT-3'	
M-6	5'-GAGGAAAGTGAGCGATGGGG-3'/5'-CTCTTCATCCCCTTCAGCCC-3'	
M-7	5'-AGCAGCCAGATTTAGAGGGG-3'/5'-ACCAGCAGACAAGTCCGAAA-3'	
M-8	5'-ACAACCCAACGAAAGACGGA-3'/5'-GTCGTGACGGCAATTTGGAC-3'	
M-10	5'-GTTCTGGAGCACCTTGAGCA-3'/5'-GCTTTGCACCTTTCTCTGCC-3'	
