# Supplementary material for: Single gametophyte sequencing reveals that crossover events differ between sexes in maize
Source: Nat Commun. 2019 Feb 15;10:785. doi: 10.1038/s41467-019-08786-x (PMC6377631; doi:10.1038/s41467-019-08786-x)
Supplement: Supplementary file 2 — Description of Additional Supplementary Data [file 41467_2019_8786_MOESM2_ESM.docx]

**Description of Additional Supplementary Files**

File Name: Supplementary Data 1: Single gametophyte sequencing data summary

Description: The basic statistics of reads mapping and SNP resolution per each sample are included.

File Name: Supplementary Data 2: CO position for male and female meiosis

Description: The detailed position and length of each CO interval detected in either embryo sac or microspore data are presented.

File Name: Supplementary Data 3: CO count per each chromosome of gametophyte (Zheng58 x SK background)

Description: The donors of these gametophyte samples are presented.

File Name: Supplementary Data 4: CO hot regions summary in gametophyte populations (Zheng58 x SK background)

Description: The 123 CO hot regions defined in female or/and male meiosis are included, with the corresponding position and cM/Mb ratio.
